# Editorial: Maternal-fetal interface formation and pregnancy outcome

**DOI:** 10.3389/fmed.2026.1751324

**Published:** 2026-02-25

**Authors:** Tao Cao, Zhengzhong Wu, Depeng Zhao

**Affiliations:** 1 Department of Reproductive Medicine, Shenzhen Maternity and Child Healthcare Hospital, Southern Medical University, Shenzhen, China; 2 Women and Children's Medical Center, Shenzhen Maternity and Child Healthcare Hospital, Southern Medical University, Shenzhen, China

**Keywords:** Endometrial Receptivity Array (ERA), inflammatory biomarkers, personalized embryo transfer, fetal intrauterine interventions, Recurrent Implantation Failure (RIF), Fetal Growth Restriction (FGR), Gestational Diabetes Mellitus (GDM), placenta previa

## Introduction

The maternal-fetal interface, the microscopic site of direct maternal-fetal communication, is the cornerstone of a successful pregnancy. It facilitates nutrient transport, waste removal, and intricate immune tolerance and endocrine regulation. Abnormalities in its establishment or maintenance can trigger a cascade of complications, including Recurrent Implantation Failure (RIF), Fetal Growth Restriction (FGR), Gestational Diabetes Mellitus (GDM), placenta previa, and postpartum hemorrhage. Therefore, understanding the mechanisms behind these abnormalities and building a comprehensive predictive and management framework is a forefront focus in modern obstetrics and gynecology.

## Endometrial receptivity abnormalities and Recurrent Implantation Failure

Successful embryo implantation depends on a brief “window of implantation,” requiring optimal endometrial receptivity (ER) (Jia et al.). ER dysfunction is a primary cause of RIF. Emerging diagnostic tools are revolutionizing this field. The receptive serum Endometrial Receptivity Test (rsERT) and Endometrial Receptivity Array (ERA) analyze endometrial gene expression profiles to pinpoint the individual implantation window. Studies show that rsERT-guided personalized embryo transfer (PET) significantly improves pregnancy outcomes in RIF patients (Li et al.). Ongoing exploration of molecular and morphological markers of ER, alongside emerging endometrial immune analysis, provides crucial insights for tailoring IVF/ICSI protocols (Liu Z. et al.). Consequently, personalized management is now feasible. For patients with PCOS, who often have compromised baseline ER, assessment becomes even more critical. The core strategy shifts from fixed-time transfers to PET guided by ERA results, coupled with exploring innovative therapies like immunomodulation to “rejuvenate” the interface.

## Pregnancy monitoring and perinatal prediction

Abnormalities at the maternal-fetal interface are reflected in fetal development and maternal pregnancy progression, making prenatal monitoring vital. Advanced imaging acts as a “barometer” for fetal wellbeing. 3D-ICRV technology measuring fetal insular volume can distinguish cortical development differences between FGR and Appropriate-for-Gestational-Age (AGA) fetuses, offering a valuable tool for prenatal assessment and counseling between 20 and 32+6 weeks (Xue et al.). Ultrasound parameters also predict delivery outcomes; multivariate logistic regression indicates that fetal Head Circumference (HC) is the most predictive factor for cesarean delivery following labor induction at 36 weeks, aiding clinical risk assessment (Liu G. et al.). Metabolic and inflammatory dysregulation signifies another facet of interface imbalance. GDM is characterized by abnormal inflammatory and immune regulators, linked to fetal organ developmental abnormalities and macrosomia. The Systemic Immune-Inflammation Index (SII) and Systemic Inflammation Response Index (SIRI) show promise as novel, non-invasive biomarkers for early identification of high-risk GDM women (Xiu et al.), enabling timely intervention and intensified monitoring. Managing complex twin pregnancies, such as Twin Anemia-Polycythemia Sequence (TAPS), exemplifies highly personalized care, requiring careful selection from options like conservative monitoring, fetoscopic laser coagulation, intrauterine transfusion, or selective reduction based on gestational age, severity, and patient preference (Zhang et al.).

## Structural abnormalities: uterine scar pregnancies

Cesarean Scar Pregnancy (CSP) is a classic structural defect at the interface, posing risks for morbidly adherent placenta and major hemorrhage. Proactive intervention is crucial for diagnosed CSP patients desiring future fertility. Early surgical management (ultrasound-guided, hysteroscopic, or laparoscopic) aims to remove gestational tissue and repair the uterine defect. Retrospective studies confirm that laparoscopic scar resection significantly reduces the recurrent cesarean scar pregnancy (RCSP) rate compared to no repair, offering an active strategy to improve subsequent pregnancy outcomes (Yin et al.).

## Postpartum complications and maternal long-term health

Postpartum management must address multiple risks comprehensively. Independent risk factors for Postpartum Hemorrhage (PPH) include ART conception, preeclampsia, placenta previa, and placental accretion, necessitating enhanced vigilance and multidisciplinary readiness (Lan et al.). For Retained Placenta (RP) without suspected abnormal invasion, expectant management is supported, as manual removal risks severe hemorrhage and hysterectomy (Ramadan et al.). Anesthesia management is also key; prophylactic ondansetron (4 mg or 8 mg) improves hemodynamic stability after spinal anesthesia for cesarean section, with only the 8 mg dose proven to reduce hypotension significantly, refining anesthetic protocols (Qin et al.). Furthermore, cesarean delivery is linked to a higher incidence of lower back pain from postpartum day 2 to week 4 compared to vaginal delivery, with high BMI and post-term pregnancy as risk factors, highlighting the need for focused postpartum recovery care (Barega et al.).

The impact of the maternal-fetal interface extends beyond the immediate postpartum period. In settings like Ethiopia, obstetric hemorrhage and hypertension remain leading causes of maternal mortality (Tesfay et al.), underscoring the need for individual and systemic interventions. A life-course perspective reveals that longer interpregnancy intervals correlate with increased risk of abdominal obesity in postmenopausal women (Su et al.), potentially mediated by sex hormone fluctuations, informing lifelong health strategies for women.

## Future perspectives and conclusion

The field is advancing toward greater precision and integration. Traditional clinical research faces challenges in recruiting patients with specific adverse outcomes and finding suitable pre-clinical models. To overcome this, Menon et al. proposed innovative virtual multi-organ system models to simulate the interface's physiology and pathology. This methodological shift, creating a “digital sandbox,” could bypass ethical constraints and accelerate the understanding of dynamic interactions, guiding intervention development. Driven by such technologies, from multi-omics to virtual modeling, and a paradigm shift toward highly personalized care—evident in ERA-guided transfers and biomarker-informed GDM management—the ultimate goal is a continuous “life-course” health management chain. This approach aims to fundamentally improve both short-term and long-term maternal and neonatal outcomes, transitioning from passively treating complications to proactively safeguarding health, reflecting the convergence of technological progress and humanistic care in modern obstetrics.

